# A Novel Plate Compartment–Confrontation Method Discovered That Volatile Organic Compounds Produced by *Saccharomyces cerevisiae* Inhibit *Botrytis cinerea* and *Fusarium graminearum*

**DOI:** 10.3390/jof11060418

**Published:** 2025-05-29

**Authors:** Ying Meng, Jing Wang, Hui Xu, Yaqi Yu, Yongheng Liang

**Affiliations:** 1 College of Life Sciences, Key Laboratory of Agricultural Environmental Microbiology of Ministry of Agriculture, Nanjing Agricultural University, Nanjing 210095, China; mying1582@163.com (Y.M.); 2023116049@stu.njau.edu.cn (J.W.); 15539120931@163.com (H.X.); 17770336278@163.com (Y.Y.); 2 Jiangsu King’s Luck Brewery Joint-Stock Co., Ltd., Lianshui 223411, China

**Keywords:** *Saccharomyces cerevisiae*, *Botrytis cinerea*, *Fusarium graminearum*, volatile organic compounds, conidiogenesis, hypha, mycelial growth, cryo-scanning electron microscopy

## Abstract

Biological control of plant diseases is important for crop production. *Botrytis cinerea* and *Fusarium graminearum* are two common pathogenic fungi which result in great harm to crop production, processing, and storage of foodstuffs. Yeasts have unique advantages to be the focus of biological control of plant diseases through multiple mechanisms, including producing volatile organic compounds (VOCs) with inhibitory effect. However, the discontinuous display of inhibitory effect by yeast VOCs on pathogenic fungi is restricted by the conventional confrontation method, and the inhibitory mechanisms are unclear. We developed a new method to detect the inhibitory effect of *Saccharomyces cerevisiae* (yeast) VOCs on *B. cinerea* and *F. graminearum*. Our results showed that the yeast VOCs inhibited the growth and development of *B. cinerea* and *F. graminearum* and the strength of the inhibitory effect is positively related to the yeast inoculation amount. We confirmed the inhibition effect of ethyl acetic, one of the main yeast VOCs, on both pathogenic fungi. We further found that the deletion or overexpression of the ethyl acetic synthesis-related genes (*ATF1* and/or *ATF2*) did not change the inhibitory effect much. The overexpression of *ATF1* changed the main composition of VOCs. One of the changed VOCs, phenethyl acetic, even had stronger inhibitory effect than ethyl acetic on *F. graminearum* when they were added alone. These results suggest that the inhibitory effect of yeast VOCs on pathogenic fungi is a complex module. The lonely added individual component of VOCs may inhibit the growth and development of pathogenic fungi, while the partial alternation of VOC composition through gene modification may not be enough to change the total inhibitory effect.

## 1. Introduction

Phytopathogens and pests present a major threat to global food security [1,2,3]. Growers worldwide lose an estimated between 10% and 23% of their crops to fungal disease every year, and another 10–20% post-harvest [2,4]. *Botrytis cinerea* and *Fusarium graminearum* are two common pathogenic fungi, which result in great harm to crop production, processing and storage of foodstuffs [5,6,7]. Therefore, it is of great significance to take effective methods to prevent crop diseases caused by *B. cinerea* and *F. graminearum*. Currently, different prevention methods, including chemical, physical, biological, and mixed methods are used to control these pathogenic fungi. Microbial and nematode-based biopesticides, as well as pathogen RNAi-based plant protection methods, are emerging [8,9,10,11]. Because of their unique advantages of no side effects on plants, non-toxic to humans and animals, and environmental friendliness, biological control methods have become ideal means to prevent and control plant diseases in recent years. Yeasts have always been the focus of biological control of plant diseases due to their unique advantages. The biocontrol mechanisms of yeasts include competing, inducing host plants to develop resistance, directly parasitizing pathogens and secreting toxins lethal to pathogenic fungus, resisting oxidative stress, forming biofilms in damaged tissues and producing inhibitory volatile organic compounds (VOCs) to inhibit pathogenic fungus [12,13,14,15,16,17,18,19,20,21,22,23].

It has been reported that the volatile and diffuse antifungal metabolites of *Pichia pastoris* and *Candida albicans* can inhibit the mycelia growth of *Sclerotinia sclerotiorum*, thus effectively preventing *S. sclerotiorum* infection in garlic [21]. The main VOCs of *Pichia anomala*, 2-phenylethanol, can inhibit the growth of *Aspergillus flavus* and the expression of aflatoxin biosynthesis genes [24]. The yeast isolate *Candida maltosa* NP9, obtained from the fermented food Iranian commercial cheese and grown on YPD agar, inhibited the spore germination of *Aspergillus brasiliensis* by VOCs [25]. The VOCs produced by *Candida interformis* C10 have a significant effect on inhibiting conidial germination and mycelium growth of *B. cinerea*. Ethyl acetate, the main VOCs of marine killing yeast, Magimage yeast, and *Saccharomyces cerevisiae*, can inhibit the growth of rot causing fungi such as *B. cinerea* and control the postharvest crop rot of strawberry [20]. Thirty-one types of *S. cerevisiae* and twenty-eight types of non-*S. cerevisiae* were able to increase enzyme activity and produce antifungal volatiles, thereby inhibiting the pathogenic fungi of sour taste and grey rot in grapes [26]. Compared with other yeast control mechanisms, there are still limited reports on yeast’s ability to produce VOCs that can inhibit plant diseases, and their mechanisms remain unclear. Therefore, it is of great significance to explore the inhibition effect of yeast VOCs on pathogenic fungi and to use yeast for biological control.

In this study, we designed an improved method to determine the confrontation effect of one microorganism to another through VOCs. The inhibitory effect could be tracked at various durations during the confrontation. We found that the VOCs produced by yeast had inhibitory effects on *B. cinerea* and *F. graminearum*, and the strength of the inhibitory effect was positively related to the yeast inoculation amount. Ethyl acetate, the main component of yeast VOCs, inhibited the growth and development of *B. cinerea* and *F. graminearum*. Alteration of ethyl acetate production through genetic methods did not change the inhibitory effect of yeast on these two fungi, although the new generated VOC phenylethyl acetate had stronger inhibition ability than ethyl acetate on *F. graminearum* if they were added alone. Therefore, the inhibitory effect of yeast VOCs on pathogenic fungi is a complex module depending not only on the composition but also on the underlying interactions among VOCs, which need further investigations.

## 2. Materials and Methods

### 2.1. Strains and Reagents

The *S. cerevisiae* yeast strains and pathogenic fungi used in this study are listed in Table 1. The primers used to delete genes and for diagnostic PCR analysis were listed in Table 2. All yeast and *Escherichia coli* transformations were performed as previously described [27]. PCR polymerases and buffers were purchased from Takara Biotechnology (Dalian, China). Commercial ethyl acetate and phenethyl acetate were purchased from Sinopharm Chemical Reagent Co., Ltd. (Shanghai, China) to verify their effect obtained with yeast VOCs.

**Figure 1:**
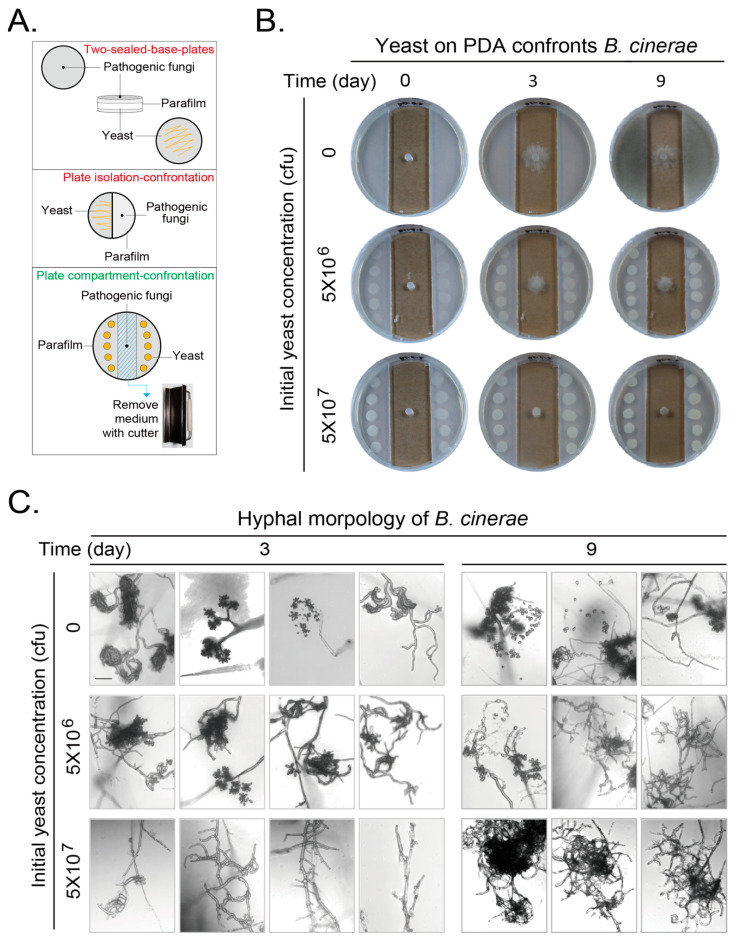
Volatile organic compounds (VOCs) produced by *Saccharomyces cerevisiae* (yeast) on PDA medium confront the growth and development of *Botrytis cinerea*. (**A**) An improved method to detect microbial confrontation by VOCs with plates. Two conventional methods: the two-sealed-base-plates method (top) and the plate isolation–confrontation method (middle). Our novel plate compartment–confrontation method. (**B**) Yeasts at the side area on PDA plates confronting growth of *B. cinerea* in middle of plate. Mature *B. cinerea* hyphae on an agar block in the middle. Yeasts at different initial amounts were placed on PDA at the side area. Growths of microbes were photographed at different durations. (**C**) Hyphal morphology of *B. cinerea* after confrontation by VOCs generated by yeast for 3 or 9 days. Plates were directly observed using a Leica laser scanning confocal microscope. Edge of *B. cinerea* hyphae was focused and taken to show representative morphologies of hyphae with or without spores. Scale bar at left top picture in corresponding panel, 100 μm. Results shown represent three independent experiments with three plates for each initial yeast amount in each experiment.

The parent *S. cerevisiae* yeast strain BY4741 was used as a wild-type (WT) strain and subjected to gene deletion to generate strains for this study. A drug-resistance cassette (*hphMX4* or *kanMX3*) was used to replace the target gene in the BY4741 strain to generate deletion strains, and selected on YPD+Hygromycin B (H8080, Solarbio, Beijing, China) and/or YPD+Geneticin (G8160, Solarbio) depending on the drug-resistance cassette. The deletion strains were further confirmed with diagnostic PCR with corresponding primers in Table 2. *ATF1* expressed from the *pUC19-PGK1pro-ATF1-PGK1t-HIS3* was constructed with the corresponding primers in Table 2 based on the fragments of pUC19, pATF1-m and pRS423 with multiple amplifications and recombination using ClonExpress ll One Step CloningKit from Vazyme Biotech (Nanjing, China). The *pUC19-PGK1pro-ATF1-PGK1t-HIS3* plasmid was linearized with NheI and integrated into the target strains [31]. The *pUC19-PGK1pro-PGK1t-HIS3* vector was used as a negative control. The strains were examined for growth phenotypes at 30 °C and for inhibitory effect on pathogenic fungi with an improved plate confrontation method as illustrated in Figure 1A. In this new method, we used only one plate by removing the medium in the middle to place the second microbe (bottom); we named this the plate compartment–confrontation method.

### 2.2. Yeast and Pathogenic Fungi Growth and Confrontation

Yeast strains were grown on rich yeast extract peptone dextrose (YPD) plates or in YPD liquid at 26 °C to prepare yeast for inoculation. YPD liquid contained 2% (*w*/*v*) peptone, 1% yeast extract, and 2% glucose. YPD plates contain 2% additional agar. An additional 0.02% Geneticin and/or 0.03% Hygromycin B were added into YPD medium to select positive colonies with gene deletion(s). Pathogenic fungi, *Botrytis cinerea* and *Fusarium graminearum* were grown on potato dextrose agar (PDA) plates at 26 °C for 2–3 days to reach 2/3 area of a plate to punch fresh hyphae at the edge for confrontation experiments. PDA plates contained 30% (*w*/*v*) diced potatoes, 2% glucose, and 2% agar. Yeast cells were inoculated onto YPD or PDA plate to confront pathogenic fungi. The overnight yeast culture was adjusted to either 5 × 10^6^ or 5 × 10^7^ cfu in 150 μL (based on 1 OD = 1 × 10^7^ cfu/mL) to inoculate onto either YPD or PDA plate at both sides with 15 μL per dot and 10 spots per plate to reach an indicated initial amount of 5 × 10^6^ or 5 × 10^7^ cfu per plate. The punched pathogenic fungi patch was located in the middle of a plate for confrontation experiment as the set-up in Figure 1A. The confrontation plates were cultured at 26 °C or 30 °C.

Different yeast cells were examined for growth phenotypes with a 10× serial dilution from approximately 5~10 OD at the first row and spotted on YPD plates to grow at 30 °C for different durations for photographing.

### 2.3. Microscopy Observation and Digital Camera Photographing

The growth plates were photographed with a digital camera (Cannon EOS 600D, Canon Inc., Tokyo, Japan) at different days for showing the inhibitory effect of yeast VOCs on pathogenic fungi. The pathogenic fungus on confrontation plates was subjected to microscopy observation or cryo-scanning electron microscopy (SEM) for hyphal morphology or conidia observation. The plates were directly subjected to observations using a Leica laser scanning confocal microscope (TCS SP8, Leica Microsystems GmbH, Wetzlar, Germany) with 20× objective lens at brightfield to focus on the edge of pathogenic hyphae to take pictures. For the hyphae with color pigments, a cellphone camera (HUAWEI Mate 9, Huawei Technologies Co., Ltd., Shenzhen, China) was applied to take color pictures through the microscope eyepiece. The confronted pathogenic fungus was cut with a blade and excessive agar medium was trimmed with the blade. The fungus mycelium patch was fixed on the sample platform with fixing solution. The samples were freeze-fixed with the rapid freezing method by liquid nitrogen mud, which was prepared with liquid nitrogen in a vacuum environment to avoid boiling the samples. The sample platform was put into the liquid nitrogen mud for about 15–25 min to quickly freeze the sample without water crystallization in the sample. The frozen fixed sample was broken through a special device under the condition of vacuum and low temperature (−100 °C) to expose a fresh section. Under −90 °C and vacuum conditions, the water wrapped in the sample was sublimed, then conductive spraying was carried out, and the sample preparation was completed. The sample was transferred to the Cryo-SEM (SU8000, Hitachi High-Tech Corporation, Tokyo, Japan) cold moving sample station (the temperature of the sample station was as low as −160 °C) for sample observation.

### 2.4. Examination and Identification for VOC Composition

The headspace–solid phase microextraction–gas chromatography–mass spectrometry (HS-SPME-GC-MS) technology was used to detect volatile substances generated by yeast cells. An Agilent 7000D Gas Chromatograph Triple Quadrupole Mass Spectrometer (GC-MS/MS) with Electron Ionization (EI) Source and National Institute of Standards and Technology (NIST) MS Data Analysis System (Agilent Technologies, Santa Clara, CA, USA) or a Comprehensive Two-Dimensional Gas Chromatography-Time-of-Flight Mass Spectrometry (GC × GC-TOF MS) System (LECO Pegasus 4D-C GC × GC-TOFMS, LECO Corporation, St. Joseph, MI, USA) in combination with an Agilent 7890A Gas Chromatograph (Agilent Technologies, Santa Clara, USA) and Time-of-Flight Mass Spectrometer (LECO Pegasus 4D TOFMS, LECO Corporation, St. Joseph, MO, USA) was used. Compared with traditional extraction technology, this technology is simpler and more convenient with higher sensitivity, shorter sample processing time, and non-organic solvents [32]. This technology was performed as previously described [24] and as follows:

(1) Yeast cells were cultured on 130 mL solid YPD agar medium in a 250 mL triangle flask (total space volume of the triangle flask is 325 mL) with amplification settings according to the agar medium volume, yeast inoculation amount, and closed empty space volume of the parameters in a petri dish, except that the space volume left was 3 times the actual space volume of the single plate. The opening of the triangle flask was closed with a silicone plug with an empty glass tube. A soft rubber hose connected to the top of the glass tube was folded and clamped to be sealed. The parafilm was used to seal all the connections to ensure that no volatile components would escape. The triangle flask was cultured for 5 or 3 days at 25 °C.

(2) The VOCs produced by yeast in the triangle flask were extracted with a polydimethylsiloxane (PDMS) solid phase microextraction head (100 μm) for about 45 min at 25 °C. After extraction, the VOCs adsorbed in the fibers were desorbed in a gas chromatography (GC) injector kept at 220 °C, injected into a DB-WAX capillary column (30 m, inner diameter 0.32 mm, film thickness 0.25 mm) (Agilent Technologies, Santa Clara, CA, USA) with helium as carrier gas.

(3) The relevant detection parameters were set as follows: 50 °C lasted for 5 min, rose to 220 °C with a temperature gradient of 4 °C/min, and extended at 220 °C for 12.5 min. The injector was used in non-shunt mode at 220 °C and the detected temperature was 230 °C. Mass spectrometer mode: 70 eV, linear retention index (LRI) was calculated for all substances using the same series of normal alkanes (C8–C20) and analyzed under the same conditions for each sample. After detection, volatile substances were identified through the National Institute of Standards and Technology (NIST) database.

## 3. Results

### 3.1. Volatile Organic Compounds (VOCs) of Yeast Confront the Growth and Development of Botrytis cinerae

The antagonism of one microbial volatile organic compounds (VOCs) to another microbe was originally detected with a two-sealed-base-plates method and later with some other methods, including the plate isolation–confrontation method [33,34] (Figure 1A top and middle). However, the limitation of the two-sealed-base-plates method is the impossible photographing at different culture durations. The media exist on the bottom of both plates, which will block the sight of both microbes for photographing. The colony morphology can be clearly photographed for one time-point only after the two plates were separated at the end of culture. Under this situation, the VOCs will be lost and the experiment is forced to stop. The plate isolation–confrontation method makes photographing at discontinuous culture duration possible. The sight block of transparent plate top can be ignored when the pictures are taken from the plate top. However, the plating of the antagonistic microbe on a half-plate is not very convenient, and the growth of the test microbe on the other half-plate will be restricted by the plate wall. Furthermore, as the agar block of the test microbe was placed on the agar medium as the antagonistic microbe. The absorbance and effect of nutrient from the fresh agar medium by the test strain cannot be excluded. We modified the plate isolation–confrontation method to be the plate compartment–confrontation method by punching out the medium from the middle bottom of the plate to locate the test microbe, while the antagonistic microbe was located on the side medium (Figure 1A, bottom). This improved method makes it possible to photograph at any culture duration without stopping the experiment. The hyphae of test strain can extend relatively freely at least at the early growth stage of the test microbe excluding the effect from the fresh side agar medium.

We first examined the effect of VOCs of yeast (BY4741) on PDA medium confronting *Botrytis cinerea* with our plate compartment–confrontation method and took growth pictures every day from 0–9 days. We only showed the growth results at the representative days. This gray mold extended its hyphae relying on the nutrient from the original agar block with visible growth at day 3 and invaded into the PDA agar medium with conidiogenesis at day 9 when no yeast was loaded onto the PDA agar. The total initial yeast amount of 5 × 10^6^ cfu per plate can obviously inhibit the mycelial growth and conidiogenesis of this gray mold at day 3 and 9. This inhibitory effect was further enhanced when the total initial yeast amount was increased to 5 × 10^7^ cfu (Figure 1B).

We observed the hyphae and conidia of *B. cinerea* at day 3 and 9 with microscopy. Without yeast, the hyphae branched and curled, and conidial peduncle could be easily observed and some spores were released at day 3. More spores were released at day 9. When yeast with initial amount of 5 × 10^6^ cfu was loaded, the conidiogenesis and spore releasing were reduced at day 3 and 9, respectively. When the initial yeast amount was further increased to 5 × 10^7^ cfu, almost no conidiogenesis and spores were observed, and the amount of curly mycelium decreased at day 3 and 9 (Figure 1C).

YPD medium is the most optimal medium for growing yeast. We also determined the growth of hyphae and conidiogenesis when yeast cells were dropped on YPD agar medium, and also observed the hyphae and conidia of *B. cinerea* at day 3 and 9 with microscopy. The results are quite similar to those obtained with yeast on PDA medium (Figure S1).

**Figure 2:**
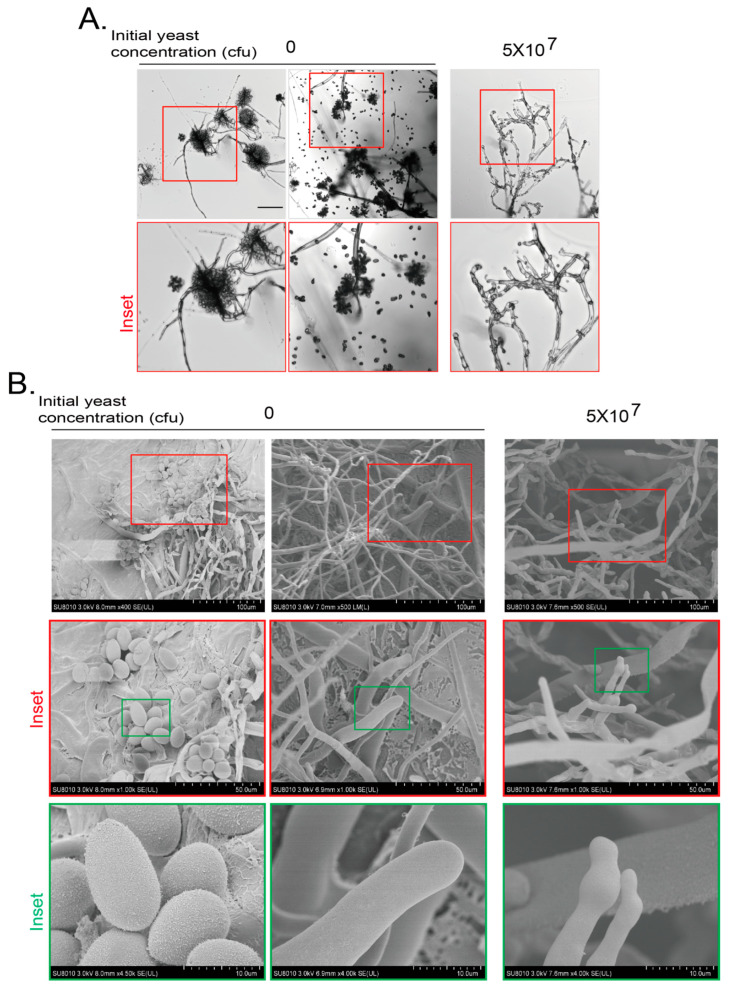
Cryo-scanning electron microscopy (SEM) observation of *B. cinerea* inhibited by VOCs of yeasts. (**A**) Hyphal morphology of *B. cinerea* after confronted by VOCs generated by yeasts by confocal microscopy. Experiments were conducted as in Figure 1B,C but with yeasts growing on PDA plates for 7 days. Inset area in the top red frame was magnified. Scale bar at left top picture, 100 μm. (**B**) Changes in hyphal morphology and conidial numbers of *B. cinerea* after confrontation by VOCs generated by yeast by cryo-SEM. Yeasts and fungi were grown as in (**A**). *B. cinerea* agar block was subjected to cryo-SEM as described in Materials and Methods. Inset areas in top red or green frames were subsequently magnified. Scale bars are shown at right bottom of each picture.

We further analyzed the details of hyphae and conidia of *B. cinerea* at day 7 with cryo-scanning electron microscopy (cryo-SEM). After the hyphae and conidia phenotypes of *B. cinerea* were confirmed with light microscopy (Figure 2A), the samples were subjected to cryo-SEM. The mycelium of *B. cinerea* at day 7 without yeast treatment were smooth and full at the tip. The oval egg-like spores were full and turgid. When the gray mold was treated with an initial yeast amount of 5 × 10^7^ cfu, spores were seldom observed and the morphology of mycelium tip became anomalous with swelling and shrinking, and some mycelium collapsed (Figure 2B). These results indicate that the VOCs from yeast inhibit the growth and development of hyphae of gray mold.

### 3.2. Volatile Organic Compounds (VOCs) of Yeast Confront the Growth and Development of Fusarium graminearum

VOCs of yeast may inhibit the growth and development of more pathogenic fungi. We further examined their effect on *F. graminearum*. The experiments were set up similarly as for the gray mold except adding one additional photographing step. As *F. graminearum* produced purple or pink pigments at the late stage of growth, the hyphae were additionally photographed with digital camera to show the color.

**Figure 3:**
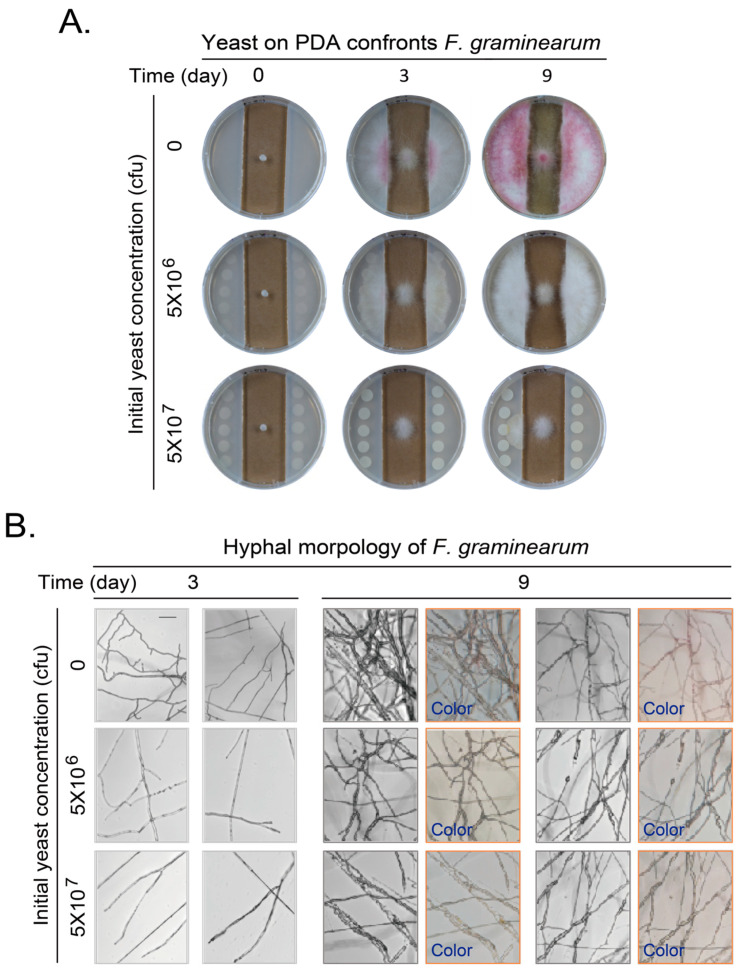
VOCs of yeast on PDA plates confronting growth and development of *Fusarium graminearum*. (**A**) Yeasts at side area on PDA plates confronting growth of *F. graminearum* in middle of plate. Experiments were performed and presented as in Figure 1B. (**B**) Hyphal morphology and color change of *F. graminearum* after confrontation by VOCs generated by yeast on PDA plates for 3 or 9 days. Experiments were performed and presented as in Figure 1C but with additional “Color” pictures taken with a cellphone camera from eyepiece of a microscope. Scale bar at left top picture in corresponding panel, 100 μm. Results shown represent three independent experiments with three plates for each initial yeast amount in each experiment.

When no yeast was loaded, *F. graminearum* gradually extended and invaded into the PDA medium at day 3. The hyphal color changed to purple at the contact site of hyphae and PDA agar medium. The hyphae expanded to the whole plate and became purple at day 9. When the initial yeast amount of 5 × 10^6^ cfu was loaded onto the PDA agar medium, the hyphae extended and invaded into the agar medium as without yeast, maybe to a lesser extent. This extension and invasion increased at day 9. However, only little or no purple hyphae can be observed. When the initial yeast amount was further increased to 5 × 10^7^ cfu, the extension and invasion decreased. There is no obvious invading into the agar medium at day 3 and only partial invading into the agar medium at day 9. No purple hyphae appeared under this situation (Figure 3A).

The hyphae of *F. graminearum* confronted by yeast VOCs were further observed with light microscopy and recorded with a cellphone digital camera. At day 3, the hyphae were at high density and with more branches when yeast was not loaded. The density of mycelium further increased and some pink pigment attached to the mycelia at day 9. When the initial yeast amount was further increased to 5 × 10^6^ or 5 × 10^7^ cfu, the hyphal branching and the density of mycelium gradually decreased as yeast amount increased. The pink or purple pigment almost disappeared (Figure 3B).

Similarly, yeasts on YPD medium also inhibited the growth and pigment formation of *F. graminearum* although the brown color of *F. graminearum* pigment on YPD medium are different from the pink or purple pigment on PDA medium (Figure S2).

**Figure 4:**
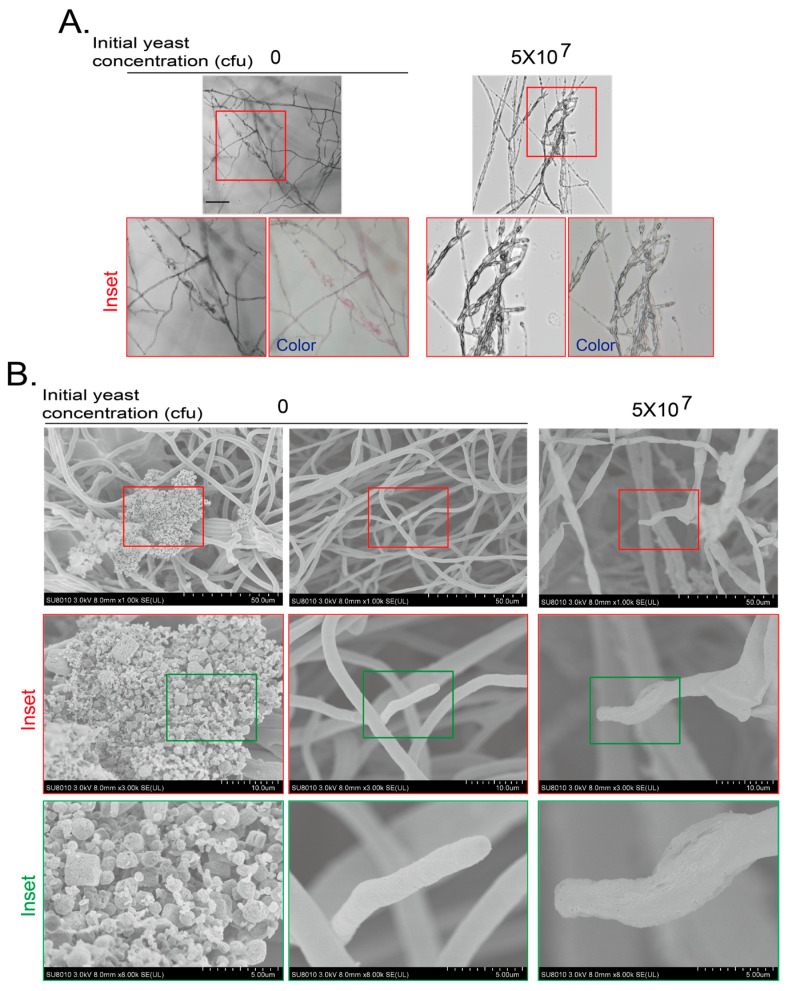
Cryo-scanning electron microscopy (SEM) observation of *F. graminearum* inhibited by VOCs of yeasts. (**A**) Hyphal morphology of *F. graminearum* confronted by VOCs generated by yeast for 7 days was observed with a confocal microscopy and a cellphone camera. Experiments were conducted as for Figure 3A,B and presented as in Figure 3B. Inset area in top red frame is magnified. Scale bar at the left top picture, 100 μm. (**B**) Hyphal morphology of *F. graminearum* confronted by VOCs generated by yeast on PDA plates for 7 days was observed by a cryo-SEM. *F. graminearum* agar block was subjected to cryo-SEM as described in Materials and Methods. Inset areas in top red or green frames were subsequently magnified.

We also applied cryo-SEM to analyze the detail of hyphae at day 7. We confirmed the hyphal morphology and color with a laser confocal microscopy and a digital cellphone camera without yeast or with a yeast initial amount of 5 × 10^7^ cfu (Figure 4A). Then, the samples were subjected to cryo-SEM. The mycelium of *F. graminearum* at day 7 without yeast treatment were smooth and full at the tip. PAD is not the favorite medium for conidiogenesis; we did not see spores. However, some irregular crystal structures could be easily observed along with the *F. graminearum* hyphae without yeast treatment at day 7, which were most likely the purple pigments. When the *F. graminearum* was treated with an initial yeast amount of 5 × 10^7^ cfu, the hyphae became wizened and collapsed (Figure 4B). These results indicate that the VOCs from yeast also inhibit the growth and pigment production of *F. graminearum*.

### 3.3. Inhibition of Growth and Development of B. cinerea and F. graminearum by One of the Yeast VOCs Ethyl Acetate

**Figure 5:**
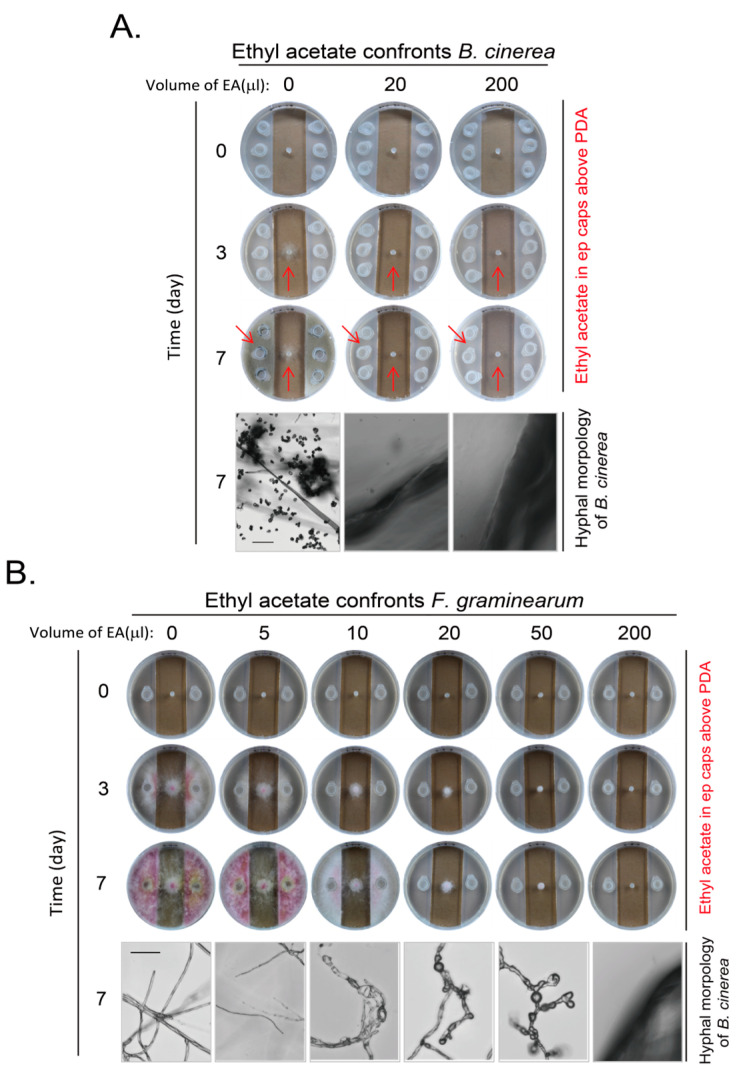
Inhibition of growth and development of *B. cinerea* and *F. graminearum* by ethyl acetate. (**A**) Ethyl acetate inhibits growth and development of *B. cinerea*. Different total volumes of ethyl acetate per plate were loaded in ep (Eppendorf) tube caps on PDA medium to imitate VOC of yeast cells, as in Figure 1B. Plates were incubated for different durations and photographed for both plates and hyphae, as indicated. Arrows emphasize area with difference for comparison. (**B**) Ethyl acetate inhibits growth and development of *F. graminearum*. Experiments were conducted as in (**A**) except that the number of Eppendorf tube cap was reduced and *F. graminearum* on an agar block was used. Scale bar at left hyphal picture in corresponding panel, 150 μm. Results shown represent three independent experiments with three plates for each ethyl acetate volume in each experiment.

In our system, yeast did not contact the fungi at least at the early stage even if the inhibition effect was not strong. They also did not share the medium for possible diffusion of metabolites through medium. The observed inhibition effects of yeast on *B. cinerea* and *F. graminearum* are through the VOCs of yeast. To further analyze the composition of the VOCs and their inhibition effect, we analyzed the VOCs of BY4741 strain through the headspace solid phase microextraction with gas chromatography–mass spectrometry (HS-SPME-GC-MS) analysis [35]. There were five major compounds in yeast VOCs in BY4741. They were ethyl acetate; ethanol; 1-butanol, 3-methyl-, acetate; 1-butanol, 3-methyl-; and phenylethyl alcohol (Figure S4). We examined whether ethyl acetate would be able to inhibit the growth and development of *B. cinerea* and *F. graminearum*. We set up an amount range of ethyl acetate with adding different volumes of ethyl acetate at a purity of more than 99.5% with density at 0.9 g/mL to examine their inhibitory effects on *B. cinerea* first. We found that 20 μL of ethyl acetate per plate was enough to inhibit the growth of *B. cinerea* at day 3 and 7. There were no hyphae at the edge of agar block of *B. cinerea* under these situations when hyphae and spores are easily seen for *B. cinerea* without loading ethyl acetate (Figure 5A). We also examined the effect of ethyl acetate on *F. graminearum* with various amounts of ethyl acetate. It is clear that ethyl acetate inhibited the growth and pigment formation of *F. graminearum* in a dose-dependent manner. A higher amount of ethyl acetate is required to inhibit hyphal growth of *F. graminearum*, as there was hyphal growth when 50 μL of ethyl acetate was applied, although the hyphae were in malformation with swelling. The hyphal growth was completely inhibited only when 200 μL of ethyl acetate was applied (Figure 5B). Therefore, the ethyl acetate in yeast VOCs might contribute to the inhibitory effect of yeast VOCs on these two pathogenic fungi.

### 3.4. Altering the Genes Regulating Ethyl Acetate Syntheis Did Not Significantly Change the Inhibitory Effect of Yeast VOCs on Pathogenic Fungi

As ethyl acetate had inhibitory effect on *B. cinerea* and *F. graminearum*, we determined whether the decreased production of ethyl acetate would reduce the inhibitory effect of yeast VOCs on these two pathogenic fungi. The alcohol acetyl transferase (AATase) is currently considered to be the key enzyme responsible to produce acetate esters (including ethyl acetate, isoamyl acetate and isobutyl acetate). *ATF1* and *ATF2* are the two key genes encoding AATase [36], and the deletion or overexpression of *ATF1* and *ATF2* can lead to decrease or increase of acetate ester contents in yeast, respectively [37,38,39,40]. When we deleted *ATF1* and/or *ATF2* from BY4741(WT) and confirmed the deletions with diagnostic PCRs (Figure S3A,B), we observed a very low reduction (between 1.2 and 2.3 times) in 3/5 of the detected VOCs, and 2/5 increased in quantity (1.4 and 1.6 times). Among them, there is a decrease but not abolishment in amount of ethyl acetate and other components (Figure S4). These deletions did not significantly reduce the growth under permissive temperature (Figure S5A). We further examined the effect of *ATF1* and *ATF2* deletion on inhibiting *B. cinerea* and *F. graminearum*. We did not observe decreased inhibitory effect by VOCs from *atf1*Δ *atf2*Δ strain on these two fungi (Figure S5B,C).

**Figure 6:**
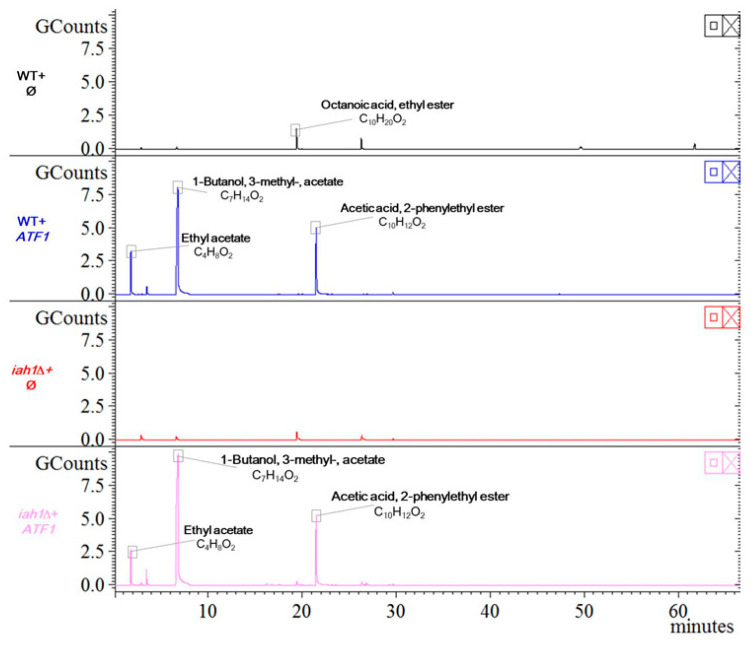
HS-SPME-GC-MS analysis for yeast VOCs from WT and *iah1*Δ strain with overexpression of *ATF1*. VOCs of indicated strains were detected by methods described in Materials and Methods. Symbol *ϕ* for vector pUC19-*PGK1pro-PGK1t-HIS3* and *ATF1* for overexpression of *ATF1* with pUC19-*PGK1pro-ATF1-PGK1t-HIS3*.

In addition, the esterase encoded by *IAH1* is one of the hydrolases that catalyzes ester bond cleavage in yeast. It has been reported that the activity of AATase could not be enhanced by removing the acetate hydrolytic esterase *Iah1*, but the cleavage of isoamyl acetate could be avoided. This way significantly increases the concentration of isoamyl acetate, and maintains an important balance between AATase and esterase, and facilitates the ester accumulation in yeast [40,41,42]. We deleted *IAH1* from BY4741(WT) and confirmed the deletion with a diagnostic PCR (Figure S3C). Therefore, overexpression of *ATF1*, especially in *iah1*Δ strain may change the ester, including ethyl acetate, accumulation greatly. There was no obvious defect in *iah1*Δ strain under permissive temperature (Figure S6A). We analyzed the VOCs in WT and *iah1*Δ strains without or with overexpression of *ATF1* and found that the octanoic acid ethyl ester almost disappeared in *iah1*Δ strain (Figure 6). In contrast, the level of ethyl acetate was drastically increased in both WT and *iah1*Δ strains with overexpression of *ATF1* in comparison to these strains without overexpression of *ATF1*. Furthermore, the overexpression of *ATF1* changed the compositions of VOCs greatly with 1-nutanol, 3-methyl-, acetate and acetic acid, 2-phenylethyl ester (alias phenethyl acetate) increasing significantly (Figure 6). However, when we applied these strains on either PDA or YPD plate to examine their effect on inhibiting *F. graminearum*, the yeast initial amount of 5 × 10^7^ cfu of different strains (WT+*ϕ*, WT+*ATF1*, *iah1*Δ+*ϕ* and *iah1*Δ+*ATF1*) on PDA for more than 3 days did not result in various inhibitory effects on inhibiting *F. graminearum*. Overexpression of *ATF1* had the same inhibitory effect on inhibiting *F. graminearum* as the control strain (Figure S6B). On YPD plates for 9 days, overexpression of *ATF1* in *iah1*Δ strain inhibited the growth and color change of *F. graminearum* slightly stronger than the other three strains (Figure S6C).

### 3.5. The New Yeast VOC Phenylethyl Acetate from Overexpression of ATF1 Inhibited Growth and Development of F. graminearum Stronger than Ethyl Acetate

**Figure 7:**
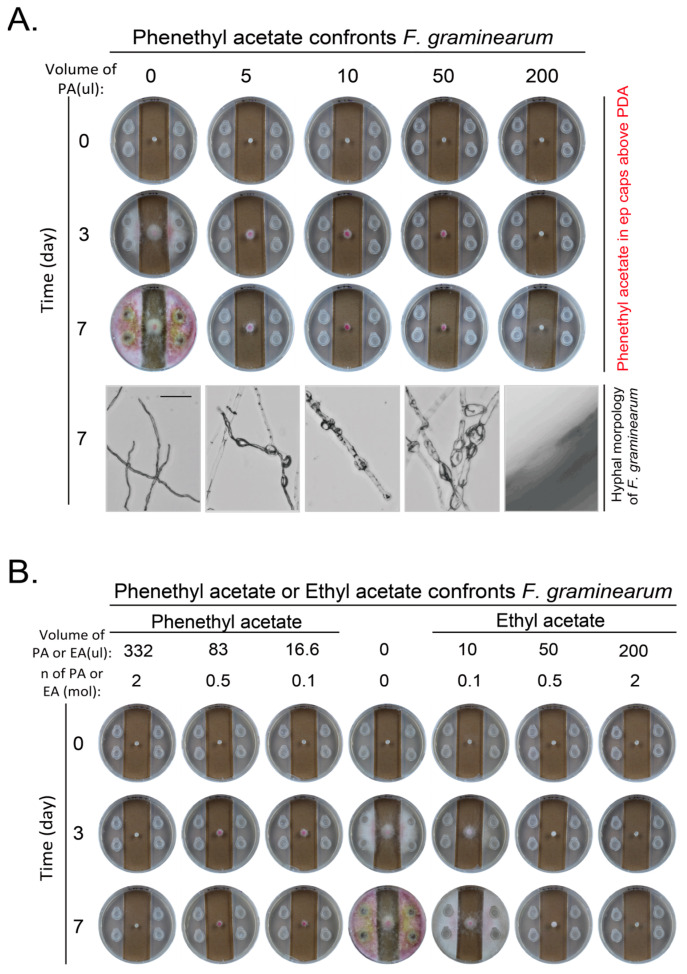
Inhibition on hyphal growth and development of *F. graminearum* by phenethyl acetate. (**A**) Phenethyl acetate inhibited growth and development of *F. graminearum*. Experiments were set up and conducted as in Figure 5B. Scale bar at the left bottom picture, 100 μm. (**B**) Phenethyl acetate inhibited growth and development of *F. graminearum* more strongly than ethyl acetate at same mole of low concentration. Same moles of phenethyl acetate and ethyl acetate were used to confront *F. graminearum* as in (**A**).

Overexpression of *ATF1* in either WT or *iah1*Δ strain did not increase the inhibitory effect on *F. graminearum* (Figure S6). However, overexpression of *ATF1* no matter in WT or *iah1*Δ strain produced new VOC compounds. The amount of 1-butanol, 3-methyl-, acetate and acetic acid, 2-phenylethyl ester (phenethyl acetate) increased most. We were curious to know whether these new components would inhibit the growth of pathogenic fungi. We found that phenethyl acetate inhibited growth and pigment production of *F. graminearum* greatly. A low amount of 5 μL of phenethyl acetate at a purity of more than 99% with density at 1.032 g/mL was enough to inhibit the growth of *F. graminearum*. The inhibitory effect gradually increased as the amount of phenethyl acetate increased. The amount of 200 μL of phenethyl acetate completely inhibited the hyphal extension and color change of *F. graminearum*. The morphology of hyphae also changed along with the increase of the amount of phenethyl acetate. The hyphae became thick and expanded until no growth when the amount of phenethyl acetate increased to 200 μL (Figure 7A). When the inhibitory effects of phenethyl acetate and ethyl acetate at the same mole concentration on *F. graminearum* were compared, phenethyl acetate had stronger inhibitory effect than ethyl acetate as 0.1 mmol of ethyl acetate did not fully inhibit the growth of *F. graminearum* while 0.1 mmol of phenethyl acetate did, although the color change was not completely inhibited (Figure 7B).

## 4. Discussion

The innovated confrontation method in this study (Figure 1A bottom) kept the advantage of the traditional two-sealed-base-plates method for avoiding direct contact between the test microbe and the antagonistic microbe through the culture medium. However, the innovated confrontation method surpassed the traditional method as the inhibitory effect can be photographed by a laser microscopy or digital camera at any culture duration, which can be done only at an end-point duration by separating the two sealed plates in the traditional method. Therefore, the innovated confrontation method makes the recording process flexible to record results at any culture duration.

The VOCs of isolated yeast cells during the processing of green coffee beans were reported to inhibit the growth of *Aspergillus* and the production of *Aspergillus* toxin through producing volatile alcohols and esters [24,43]. In addition, it has been reported that the main volatile substance of *Wickerhamomyces anomalus*, *Metschnikowia pulcherrima* and *S. cerevisiae* is ethyl acetate [20], and the main volatile substances of *Candida intermedia* are 1,3,5,7-cyclooctatetraene and 3-methyl-1-butanol [19]. We found that the VOCs of *S. cerevisiae* inhibited the growth and development of two pathogenic fungi in this study, including conidiogenesis, pigment production, and hyphae malformation (Figure 1, Figure 2, Figure 3 and Figure 4 and Figure S1, Figure S2, Figure S5 and Figure S6). The most obvious phenotypes of studied phytopathogens by yeast VOCs or components on plates are colony size and pigment production. Pigment production is closely related to fungus growth and sporulation, and usually appears as a secondary product at the later growth stage for UV protection, spore dispersal, and other functions. Their suppression could weaken the fungus’s ability to colonize hosts or withstand environmental stressors [44,45]. We observed dark color for *B. cinerea* and pink color for *F. graminearum* on both hyphae and culture medium when there was no or less inhibition. In contrast, there was no pigment production when yeast initial inoculation or VOC component amount increased. Exposure to VOCs from yeast was also observed to cause changes in pigmentation of other fungi [46]. We do not know exactly why yeast VOCs and components inhibit pigment production of *B. cinerea* and *F. graminearum*; in this study, there are some proposed mechanisms for the influence of VOCs on the pigment production, including interference with metabolic pathways, stress responses, and microbial competition. Ethanol and 2-phenylethanol, identified in yeast VOC profiles, are known to impair membrane integrity and mitochondrial function, potentially disrupting pigment precursor synthesis. We may explore the detailed mechanisms in the future. These inhibitions are highly depending on yeast initial inoculation amount, which determines yeast VOC concentration. Suppression of pathogenic pigments by yeast VOCs suggests a novel biocontrol strategy to mitigate crop diseases. The effect of the yeast culture medium could be ignored as the test fungus strain did not have chance to touch the yeast culture medium if the hyphal growth was inhibited. VOCs of yeast culture medium could also be ignored as the negative control without inoculating yeast did not show phenotypes caused by yeast inoculation (Figure 1, Figure 2, Figure 3 and Figure 4).

We determined the VOCs of yeast and found there were a few major components, (including ethyl acetate and 1-butanol, 3-methyl-, acetate) (Figure S4). The ethyl acetate was reported to be the most important component of volatile substances of *S. cerevisiae*. Our results are consistent with those reported in the literature [19]. Ethyl acetate was reported to inhibit *B. cinerea* [20]. We found that the amount of ethyl acetate required to inhibit *B. cinerea* in this study was lower (Figure 5A). We also found a strong inhibitory effect of ethyl acetate on *F. graminis* for growth and development in this study. Therefore, the ethyl acetate in the yeast VOCs most likely contributed to the inhibitory effect on pathogenic fungi. We did not examine the effect of other single VOC component on the inhibitory effect on these two pathogenic fungi. We also did not examine the effect of different combinations of the VOC components on these two pathogenic fungi. The effect of these conditions needs to be examined in the future.

As the effect of ethyl acetate in yeast VOCs on inhibiting pathogenic fungi was confirmed in different fungal species from different labs, we tried to change the level of ethyl acetate through genetic alternations to adjust the inhibitory effect. We deleted the ethyl acetate synthesis related genes *ATF1* and *ATF2*, and observed the decrease in content of ethyl acetate and others (Figure S4) as reported [37,38,39,40]. However, we did not observe a decreased inhibitory effect for yeast VOCs on *B. cinerea* and *F. graminearum*. We also overexpressed *ATF1* in WT and *iah1*Δ strains, and noticed that the ethyl acetate amount increased, and two other components (1-butanol, 3-methyl-, acetate and acetic acid, 2-phenylethyl ester (phenethyl acetate)) significantly increased. The new VOC phenethyl acetate inhibited *F. graminearum* even more strongly than ethyl acetate. In these examinations, the results from yeast VOCs and from the pure VOC chemical are inconsistent. When the pure VOC chemical showed inhibitory effect, the changes in yeast VOCs by genetic alternations did not show the expected results, indicating the composition of VOC components is more important than a single VOC component in inhibiting pathogenic fungi. This is also important to pay attention to in similar future studies that the results from a pure chemical may not be equivalent to the results from a change of that chemical in a mixture.

We clarified the VOC component from WT or *iah1*Δ strains with overexpression of *ATF1*. However, we still did not answer the mechanism of inhibitory effect from yeast VOCs or single VOC component. These can be partially answered with proteomics and genomics studies of the antagonistic fungus in the future.

## 5. Conclusions

By using an improved confrontation detection method, we showed the inhibitory effect of yeast VOCs on the changes of hyphal morphology and development of *B. cinerea* and *F. graminearum*. Two of the commercial VOC components, ethyl acetate and phenethyl acetate, also had obvious inhibition effect on these two pathogenic fungi. However, the alternation of yeast VOC components by deleting or overexpressing ethyl acetate synthesis-related genes had limited effect on changing the inhibitory effect on the growth and development of *B. cinerea* and *F. graminearum*. We also did not examine the inhibition effect of other VOC components. Therefore, the VOC components with dominant inhibition effect and the contradictory results from genetically modified yeast for VOCs need to be further investigated so that the underlying mechanism can be finally resolved in the future.

## Figures and Tables

**Table 1:** *S. cerevisiae* yeast strains and pathogenic fungi used in this study.

Strain Number	Alias	Genotype	Source
YLY2323	BY4741	*MAT a his3* *∆* *1 leu2* *∆* *0 met15* *∆* *0 ura3* *∆* *0*	[28]
YLY4049		YLY2323 *atf1∆::kanMX3*	This study
YLY4050		YLY2323 *atf2∆::kanMX3*	This study
YLY4053		YLY2323 *atf1∆::hphMX4 atf2∆::kanMX3*	This study
YLY4055		YLY2323 *iah∆::kanMX3*	This study
	B05.10	*Botrytis cinerea*	[29]
	PH-1	*Fusarium graminearum*	[30]

**Table 2:** Primers used for deletion, diagnostic PCR, or plasmid construction in this study.

Primer #	Primer Name	Sequence
YLO-2821	*ATF1*+500 For	TGACACCCGGATAATTAAGAAGTGG
YLO-2822	*ATF1*+500 Rev	GGGAGAAGTCCGAAAAATGCGTATC
YLO-877	Ptef	ACCCATGGTTGTTTATGTTC
YLO-2403	*ATF1::Hyg* For	ATCACAAATACCATCAATTTATCAGCTCTCATGAATGAAAGATCTGTTTAGC TTGCCTC
YLO-2404	*ATF1::Hyg* Rev	GAATAATATCAGTCAAGCATCATGTGAGATCTAAGGGCCTGCTCGTTTTCGACACTGGG
YLO-2823	*ATF1::KanMX* For	ATCACAAATACCATCAATTTATCAGCTCTCATGAATGAACCAAAACTAACCAGCTGAAGCTTCGTACGC
YLO-2824	*ATF1::KanMX* Rev	GAATAATATCAGTCAAGCATCATGTGAGATCTAAGGGCCTTAGGCCACTAGT GGATCTG
YLO-2396	*ATF1* Diag For	GGGCGACAGTATTTCAAGAC
YLO-2825	*ATF2::KanMX* For	CTTCAGCAATAAAAATTGTCCAGGTTAATTCCAAAACTAACCAGCTGAAGCTTCGTACGC
YLO-2826	*ATF2::KanMX* Rev	TATACGAAGGCCCGCTACGGCAGTA TCGCATAGGCCACTAGTGGATCTG
YLO-2395	*ATF2* Diag For	CCGATGGGAGGTCCATCGGC
YLO-2827	*IAH1::KanMX* For	TCTGTTCGTACGCTTAAACTGTGACCAAATCCAAAACTAACCAGCTGAAGCTTCGTACGC
YLO-2828	*IAH1::KanMX* Rev	AGACAGAGTACGTACAAAGGATTA CTGCATTAGGCCACTAGTGGATCTG
YLO-2531	*IAH1* Diag up	TTTTTTCTGGGAGGACTGCAGAAGCT GAGA
YLO-2523	F-SmaI-HIS3p	GTGAATTCGAGCTCGGTACCCGGGCCCGGGCTAGTACACTCTATATTTTT
YLO-2524	R-SmaI-HIS3	CCTGCAGGTCGACTCTAGAGGATCCCCGGGCTACATAAGAACA
YLO-2542	F-PGK1p-XhoI-ATF1	ACTTTTTACAACAAATATAAAACACTCGAGATGAATGAAATCGATGAGAA
YLO-2543	R-PGK1t-XhoI-ATF1	CTATCGATTTCAATTCAATTCAATCTCGAGCTAAGGGCCTAAAAGGAGAG

## Data Availability

The original contributions presented in this study are included in the article/Supplementary Materials. Further inquiries can be directed to the corresponding author.

## Supplementary Materials

The following supporting information can be downloaded at: https://www.mdpi.com/article/10.3390/jof11060418/s1, Figure S1: Volatile organic components (VOCs) of yeast on YPD plates confront growth and development of *Botrytis cinerae*; Figure S2: VOCs of yeast on YPD plates confront growth and development of *F. graminearum*; Figure S3: Diagnostic PCR to confirm deletions in deletion candidate strains; Figure S4: HS-SPME-GC-MS analysis for yeast VOCs from BY4741 (WT) and *atf1*Δ *atf2*Δ strains. Figure S5: Double deletion of *ATF1* and *ATF2* did not result in significant decrease of inhibitory effect of yeast VOCs on pathogenic fungi; Figure S6: Detection of inhibition of *F. graminearum* by yeast VOCs from WT and *iah1*Δ strain with overexpression of *ATF1*.

## References

[1] Zhang Q.Q., Men X.Y., Hui C., Ge F., Ouyang F. (2022). Wheat yield losses from pests and pathogens in China. Agric. Ecosyst. Environ..

[2] Stukenbrock E., Gurr S. (2023). Address the growing urgency of fungal disease in crops. Nature.

[3] Fones H.N., Bebber D.P., Chaloner T.M., Kay W.T., Steinberg G., Gurr S.J. (2020). Threats to global food security from emerging fungal and oomycete crop pathogens. Nat. Food.

[4] Steinberg G., Gurr S.J. (2020). Fungi, fungicide discovery and global food security. Fungal Genet. Biol..

[5] Rosslenbroich H.-J., Stuebler D. (2000). *Botrytis cinerea*—History of chemical control and novel fungicides for its management. Crop Prot..

[6] Williamson B., Tudzynski B., Tudzynski P., van Kan J.A. (2007). *Botrytis cinerea*: The cause of grey mould disease. Mol. Plant Pathol..

[7] Sarrocco S., Esteban P., Vicente I., Bernardi R., Plainchamp T., Domenichini S., Puntoni G., Baroncelli R., Vannacci G., Dufresne M. (2021). Straw competition and wheat root endophytism of *Trichoderma gamsii* T6085 as useful traits in the biological control of Fusarium Head Blight. Phytopathology.

[8] Hamrouni R., Regus F., Farnet Da Silva A.M., Orsiere T., Boudenne J.L., Laffont-Schwob I., Christen P., Dupuy N. (2025). Current status and future trends of microbial and nematode-based biopesticides for biocontrol of crop pathogens. Crit. Rev. Biotechnol..

[9] Sellamuthu G., Chakraborty A., Vetukuri R.R., Sarath S., Roy A. (2024). RNAi-biofungicides: A quantum leap for tree fungal pathogen management. Crit. Rev. Biotechnol..

[10] Wytinck N., Manchur C.L., Li V.H., Whyard S., Belmonte M.F. (2020). dsRNA Uptake in Plant Pests and Pathogens: Insights into RNAi-Based Insect and Fungal Control Technology. Plants.

[11] Septiani P., Pramesti Y., Ghildan M., Aprilia K.Z., Awaludin R., Medina S., Subandiyah S., Meitha K. (2025). RNAi-based biocontrol for crops: A revised expectation for a non-recent technology. Planta.

[12] Bencheqroun S.K., Bajji M., Massart S., Bentata F., Labhilili M., Achbani H., El Jaafari S., Jijakli M.H. (2006). Biocontrol of blue mold on apple fruits by *Aureobasidium pullulans* (strain Ach 1-1): In vitro and in situ evidence for the possible involvement of competition for nutrients. Commun. Agric. Appl. Biol. Sci..

[13] Wang X.X., Chi Z., Peng Y., Wang X.H., Ru S.G., Chi Z.M. (2012). Purification, characterization and gene cloning of the killer toxin produced by the marine-derived yeast *Williopsis saturnus* WC91-2. Microbiol. Res..

[14] Xu B., Zhang H., Chen K., Xu Q., Yao Y., Gao H. (2013). Biocontrol of postharvest Rhizopus decay of peaches with *Pichia caribbica*. Curr. Microbiol..

[15] Castoria R., Caputo L., De Curtis F., De Cicco V. (2003). Resistance of postharvest biocontrol yeasts to oxidative stress: A possible new mechanism of action. Phytopathology.

[16] Lutz M.C., Lopes C.A., Rodriguez M.E., Sosa M.C., Sangorrin M.P. (2013). Efficacy and putative mode of action of native and commercial antagonistic yeasts against postharvest pathogens of pear. Int. J. Food Microbiol..

[17] Haissam J.M. (2011). *Pichia anomala* in biocontrol for apples: 20 years of fundamental research and practical applications. Antonie Leeuwenhoek.

[18] Sui Y., Liu J., Wisniewski M., Droby S., Norelli J., Hershkovitz V. (2012). Pretreatment of the yeast antagonist, *Candida oleophila*, with glycine betaine increases oxidative stress tolerance in the microenvironment of apple wounds. Int. J. Food Microbiol..

[19] Huang R., Li G.Q., Zhang J., Yang L., Che H.J., Jiang D.H., Huang H.C. (2011). Control of postharvest Botrytis fruit rot of strawberry by volatile organic compounds of *Candida intermedia*. Phytopathology.

[20] Oro L., Feliziani E., Ciani M., Romanazzi G., Comitini F. (2018). Volatile organic compounds from *Wickerhamomyces anomalus*, *Metschnikowia pulcherrima* and *Saccharomyces cerevisiae* inhibit growth of decay causing fungi and control postharvest diseases of strawberries. Int. J. Food Microbiol..

[21] Cavalcanti V.P., Araújo N.A.F., Machado N.B., Costa P.S.P., Pasqual M., Alves E., Schwan-Estrada K.R.F., Dória J. (2020). Yeasts and *Bacillus* spp. as potential biocontrol agents of *Sclerotinia sclerotiorum* in garlic. Sci. Hortic..

[22] Liu J., Wisniewski M., Droby S., Vero S., Tian S., Hershkovitz V. (2011). Glycine betaine improves oxidative stress tolerance and biocontrol efficacy of the antagonistic yeast *Cystofilobasidium infirmominiatum*. Int. J. Food Microbiol..

[23] Medina-Córdova N., López-Aguilar R., Ascencio F., Castellanos T., Campa-Córdova A.I., Angulo C. (2016). Biocontrol activity of the marine yeast *Debaryomyces hansenii* against phytopathogenic fungi and its ability to inhibit mycotoxins production in maize grain (*Zea mays* L.). Biol. Control.

[24] Hua S.S., Beck J.J., Sarreal S.B., Gee W. (2014). The major volatile compound 2-phenylethanol from the biocontrol yeast, *Pichia anomala*, inhibits growth and expression of aflatoxin biosynthetic genes of *Aspergillus flavus*. Mycotoxin Res..

[25] Ando H., Hatanaka K., Ohata I., Yamashita-Kitaguchi Y., Kurata A., Kishimoto N. (2012). Antifungal activities of volatile substances generated by yeast isolated from Iranian commercial cheese. Food Control.

[26] Nally M.C., Pesce V.M., Maturano Y.P., Assaf L.A.R., Toro M.E., de Figueroa L.I.C., Vazquez F. (2015). Antifungal modes of action of and other biocontrol yeasts against fungi isolated from sour and grey rots. Int. J. Food Microbiol..

[27] Liang Y., Morozova N., Tokarev A.A., Mulholland J.W., Segev N. (2007). The role of Trs65 in the Ypt/Rab guanine nucleotide exchange factor function of the TRAPP II complex. Mol. Biol. Cell.

[28] Brachmann C.B., Davies A., Cost G.J., Caputo E., Li J., Hieter P., Boeke J.D. (1998). Designer deletion strains derived from *Saccharomyces cerevisiae* S288C: A useful set of strains and plasmids for PCR-mediated gene disruption and other applications. Yeast.

[29] Ren W., Zhang Z., Shao W., Yang Y., Zhou M., Chen C. (2017). The autophagy-related gene *BcATG1* is involved in fungal development and pathogenesis in *Botrytis cinerea*. Mol. Plant Pathol..

[30] Wang M., Wu L., Mei Y., Zhao Y., Ma Z., Zhang X., Chen Y. (2020). Host-induced gene silencing of multiple genes of *Fusarium graminearum* enhances resistance to Fusarium head blight in wheat. Plant Biotechnol. J..

[31] Xie Z., Nair U., Klionsky D.J. (2008). Atg8 controls phagophore expansion during autophagosome formation. Mol. Biol. Cell.

[32] Fan W., Qian M.C. (2005). Headspace solid phase microextraction and gas chromatography-olfactometry dilution analysis of young and aged Chinese “Yanghe Daqu” liquors. J. Agric. Food Chem..

[33] Dennis C., Webster J. (1971). Antagonistic properties of species-groups of *Trichoderma*. Trans. Br. Mycol. Soc..

[34] Alvarez-Garcia S., Mayo-Prieto S., Carro-Huerga G., Rodriguez-Gonzalez A., Gonzalez-Lopez O., Gutierrez S., Casquero P.A. (2021). Volatile organic compound chamber: A novel technology for microbiological volatile interaction assays. J. Fungi.

[35] Lancioni C., Castells C., Candal R., Tascon M. (2022). Headspace solid-phase microextraction: Fundamentals and recent advances. Adv. Sample Prep..

[36] Holt S., Trindade de Carvalho B., Foulquie-Moreno M.R., Thevelein J.M. (2018). Polygenic Analysis in Absence of Major Effector ATF1 Unveils Novel Components in Yeast Flavor Ester Biosynthesis. mBio.

[37] Lilly M., Lambrechts M.G., Pretorius I.S. (2000). Effect of increased yeast alcohol acetyltransferase activity on flavor profiles of wine and distillates. Appl. Environ. Microbiol..

[38] Zhang J., Zhang C., Qi Y., Dai L., Ma H., Guo X., Xiao D. (2014). Acetate ester production by Chinese yellow rice wine yeast overexpressing the alcohol acetyltransferase-encoding gene *ATF2*. Genet. Mol. Res..

[39] Fujii T., Yoshimoto H., Tamai Y. (1996). Acetate ester production by *Saccharomyces cerevisiae* lacking the *ATF1* gene encoding the alcohol acetyltransferase. J. Ferment. Bioeng..

[40] Li W., Wang J.-H., Zhang C.-Y., Ma H.-X., Xiao D.-G. (2017). Regulation of *Saccharomyces cerevisiae* genetic engineering on the production of acetate esters and higher alcohols during Chinese Baijiu fermentation. J. Ind. Microbiol. Biotechnol..

[41] Ma J., Lu Q., Yuan Y., Ge H., Li K., Zhao W., Gao Y., Niu L., Teng M. (2010). Crystal structure of isoamyl acetate-hydrolyzing esterase from *Saccharomyces cerevisiae* reveals a novel active site architecture and the basis of substrate specificity. Proteins Struct. Funct. Bioinform..

[42] Fukuda K., Yamamoto N., Kiyokawa Y., Yanagiuchi T., Wakai Y., Kitamoto K., Inoue Y., Kimura A. (1998). Balance of activities of alcohol acetyltransferase and esterase in *Saccharomyces cerevisiae* is important for production of isoamyl acetate. Appl. Environ. Microbiol..

[43] Masoud W., Poll L., Jakobsen M. (2005). Influence of volatile compounds produced by yeasts predominant during processing of *Coffea arabica* in East Africa on growth and ochratoxin A (OTA) production by *Aspergillus ochraceus*. Yeast.

[44] Bölker M., Basse C.W., Schirawski J. (2008). *Ustilago maydis* secondary metabolism-from genomics to biochemistry. Fungal Genet. Biol..

[45] Demain A.L., Fang A. (2000). The natural functions of secondary metabolites. Adv. Biochem. Eng. Biotechnol..

[46] Bruce A., Stewart D., Verrall S., Wheatley R.E. (2003). Effect of volatiles from bacteria and yeast on the growth and pigmentation of sapstain fungi. Int. Biodeterior. Biodegrad..

