# Supplementary material for: A Novel Plate Compartment–Confrontation Method Discovered That Volatile Organic Compounds Produced by Saccharomyces cerevisiae Inhibit Botrytis cinerea and Fusarium graminearum
Source: J Fungi (Basel). 2025 May 29;11(6):418. doi: 10.3390/jof11060418 (PMC12193890; doi:10.3390/jof11060418)
Supplement: Supplementary file 1 [file jof-11-00418-s001.zip › jof-3628778-supplementary.pdf]

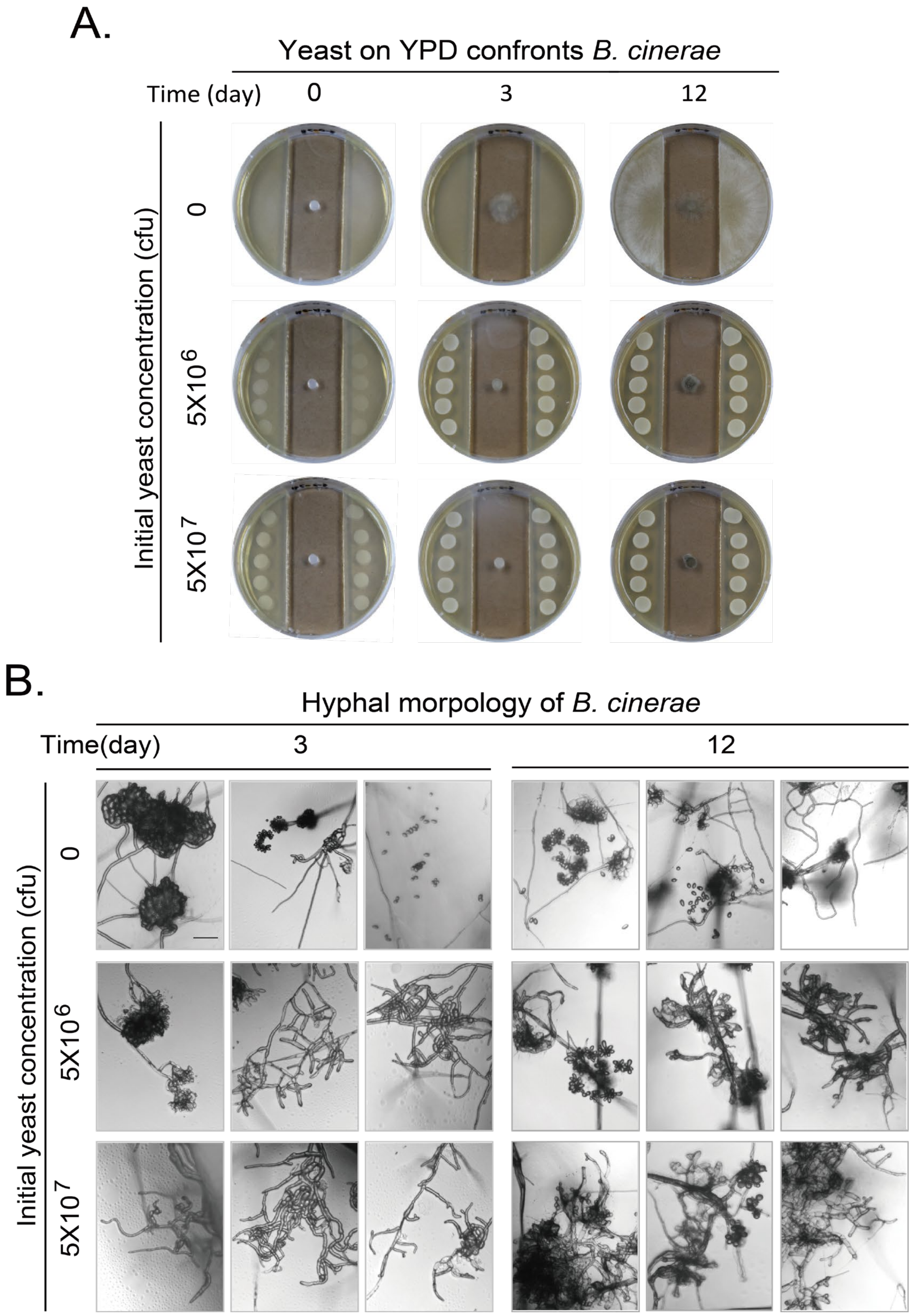

Figure S1. Volatile organic components (VOCs) of yeast on YPD plates confronting growth and development of *Botrytis cinerea*. The experiments were set up and presented as in Figure 1B-C except that PDA medium was replaced with YPD medium in this figure. (**A**) Yeasts at side area on YPD plates confronting growth of *B. cinerea* in middle of plate. (**B**) Hyphal morphology of *B. cinerea* after confrontation by VOCs generated by yeast of different initial amounts growing on YPD plates for 3 or 12 days. Scale bar at left top picture in corresponding panel, 100 μm. Results shown represent three independent experiments with three plates for each yeast initial amount in each experiment.

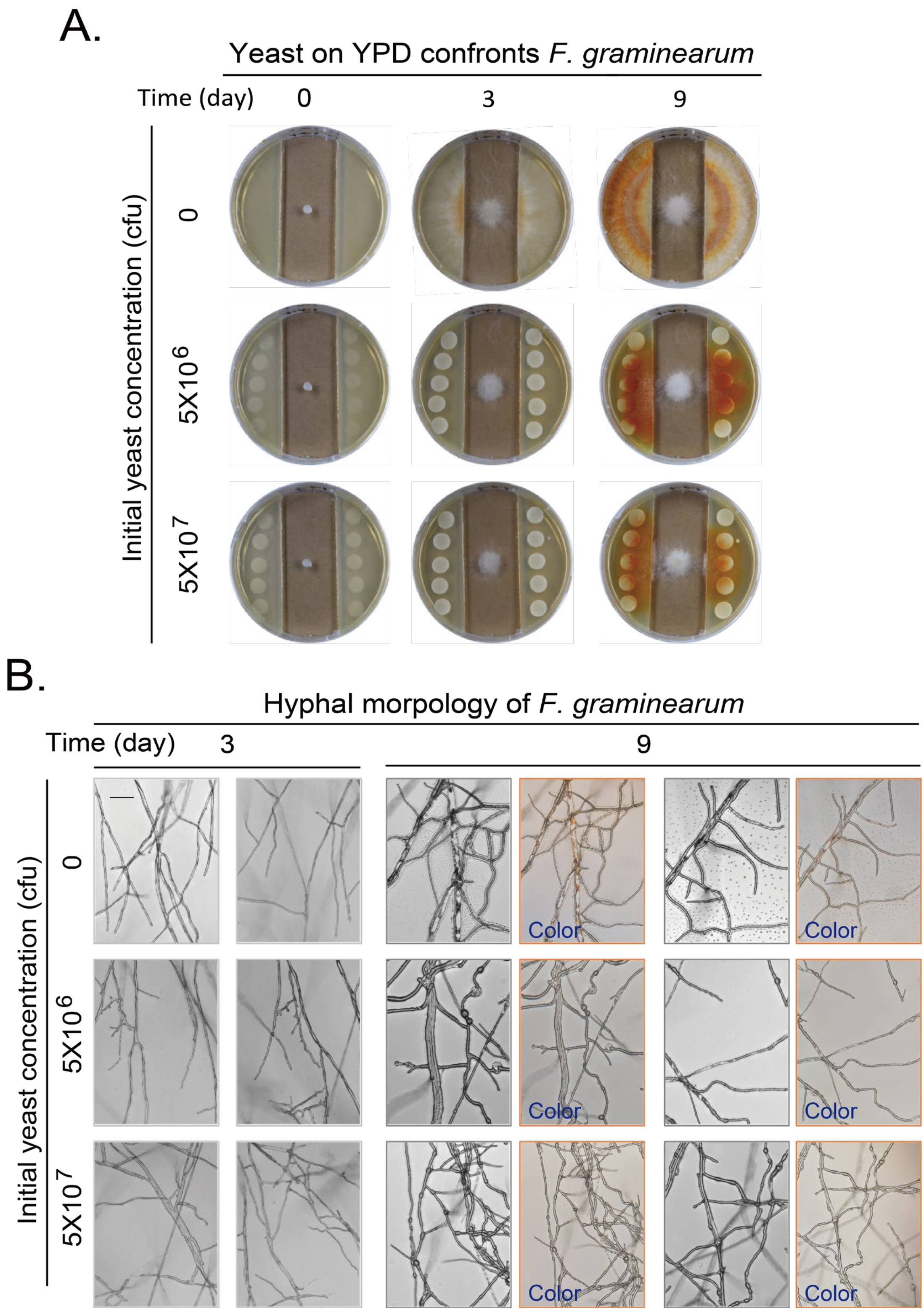

Figure S2. VOCs of yeast on YPD plates confronting growth and development of *F. graminearum*. Experiments were set up and presented as in Figure 3A-B except that PDA medium was replaced with YPD medium in this figure. (**A**) Yeasts at side area on YPD plates confronting growth of *F. graminearum* in middle of plate. (**B**) Hyphal morphology and color change of *F. graminearum* after confrontation by VOCs generated by yeast of different initial amounts growing on YPD plates for 3 or 9 days. Pictures were taken and shown as in Figure 3B. Scale bar at left top picture in corresponding panel, 100 μm. Results shown represent three independent experiments with three plates for each yeast initial amount in each experiment.

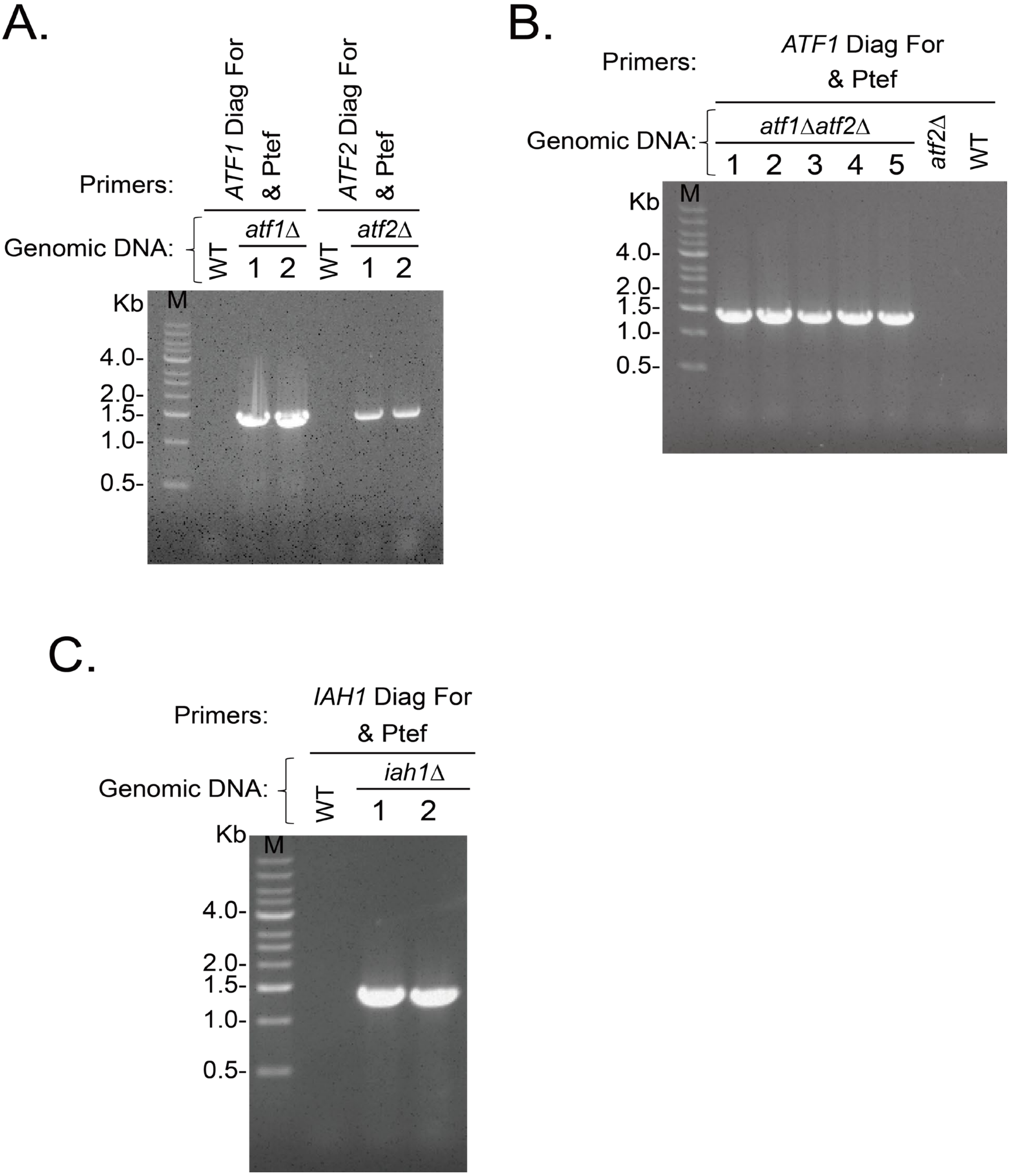

Figure S3. Diagnostic PCR to confirm deletions in deletion candidate strains. (**A**) Diagnostic PCR to confirm *atf1*Δ and *atf2*Δ candidate strains with *kanMX3* as a deletion cassette. (**B**) Diagnostic PCR to confirm *atf1*Δ*atf2*Δ candidate strains with *hphMX4* as a deletion cassette to delete *ATF1* from *kanMX3* deleted *ATF2* strains. (**C**) Diagnostic PCR to confirm *iah1*Δ candidate strains with *kanMX3* as a deletion cassette. Negative strains were included for comparison. M for DNA ladder.

A.

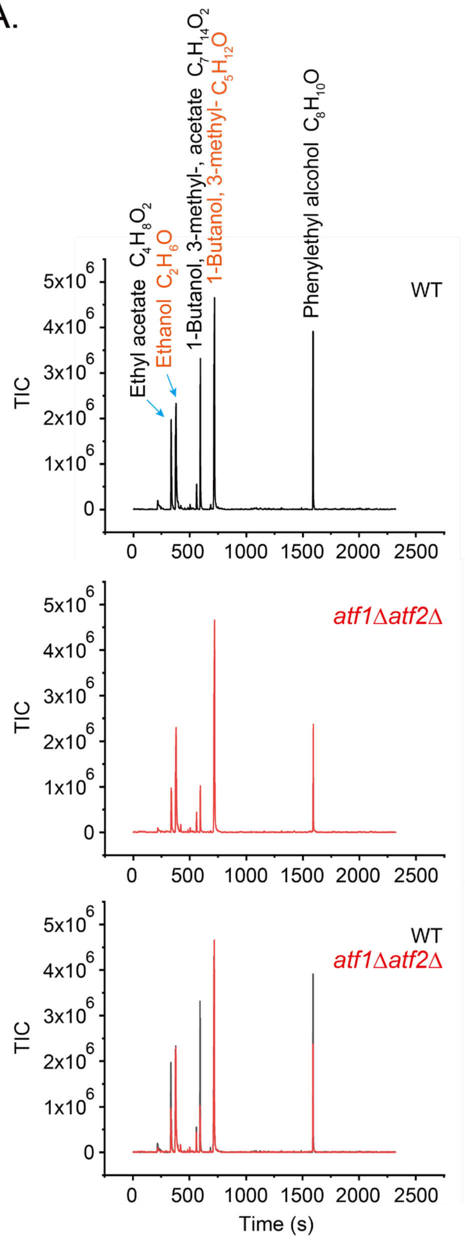

B.

Name	R.T. (s)	Area		Area %		CAS
		WT	*atf1Δatf2Δ*	WT	*atf1Δatf2Δ*	
Ethyl acetate	335.3	80368317	39713827	**24.439**	17.947	141-78-6
Ethanol	378.7	44363388	43486268	13.49	19.651	64-17-5
1-Butanol, 3-methyl-, acetate	593.2	54397033	16001526	**16.541**	7.2311	123-92-2
1-Butanol, 3-methyl-	718.2	39343671	41442851	11.964	18.728	123-51-3
Phenylethyl alcohol	1592.2	36572398	19998719	**11.121**	9.0374	60-12-8

Figure S4. HS-SPME-GC-MS analysis for yeast VOCs from BY4741 (WT) and *atf1Δatf2Δ* strains. VOCs were similarly detected by methods described in materials and methods but using total ion chromatogram (TIC) as readouts. (**A**) TICs for yeast VOCs from BY4741 (WT) (top), *atf1Δatf2Δ* (middle) and both (bottom) strains. Major TIC peaks were identified and marked for WT. (**B**) Identification of peaks for panel A.

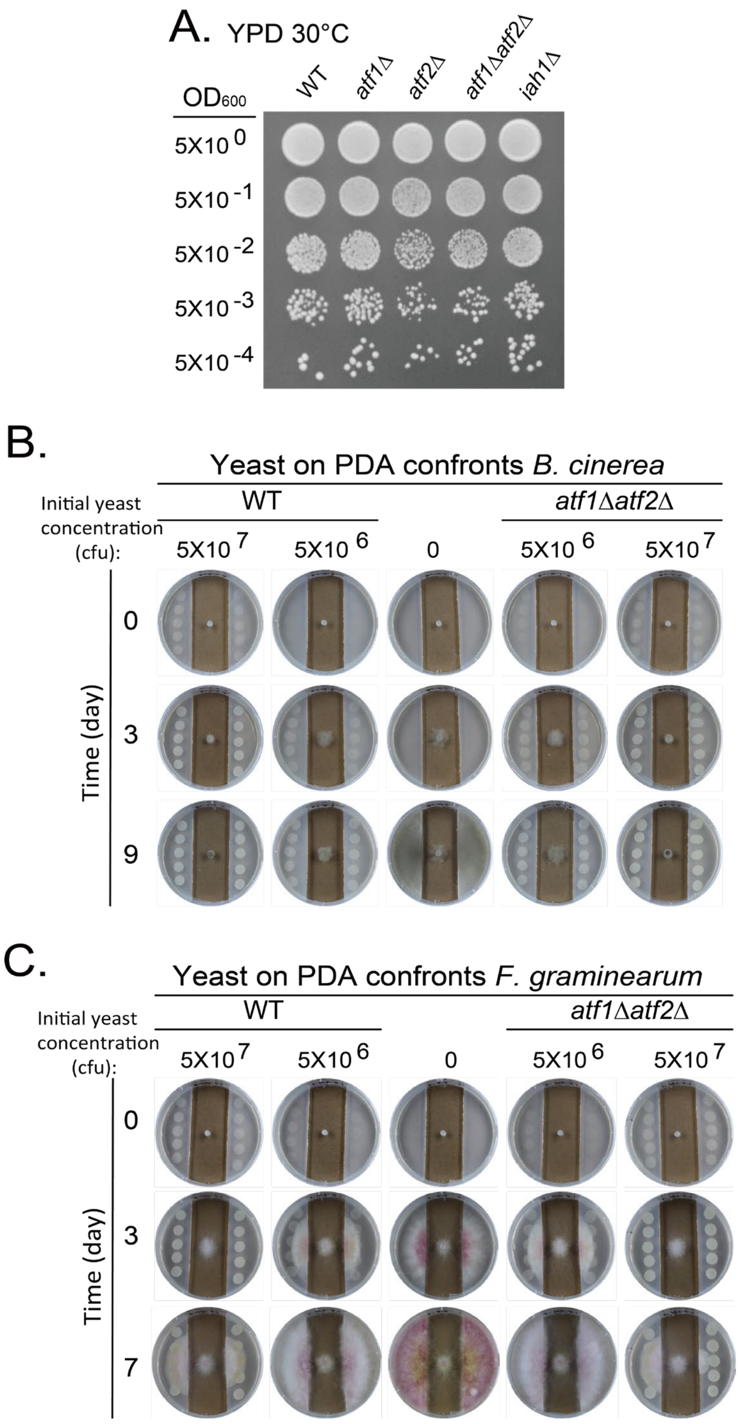

Figure S5. Double deletion of *ATF1* and *ATF2* did not result in significant decrease of inhibitory effect of yeast VOCs on pathogenic fungi. (**A**) There was no growth defect for indicated mutants on YPD at 30°C. Yeast cells at approximately 5 OD at the first row were serially diluted with a 10 × dilution. (**B**) Inhibitory effect on growth and differentiation of *B. cinerea* was not reduced in *atf1*Δ*atf2*Δ strain on PDA medium. Experiments were set up as in Figure 1B. (**C**) Inhibitory effect on growth and differentiation of *F. graminearum* was not reduced in *atf1*Δ*atf2*Δ strain on PDA medium. Experiments were set up as in Figure 3A. Results shown represent three independent experiments with three plates for each yeast initial amount in each experiment.

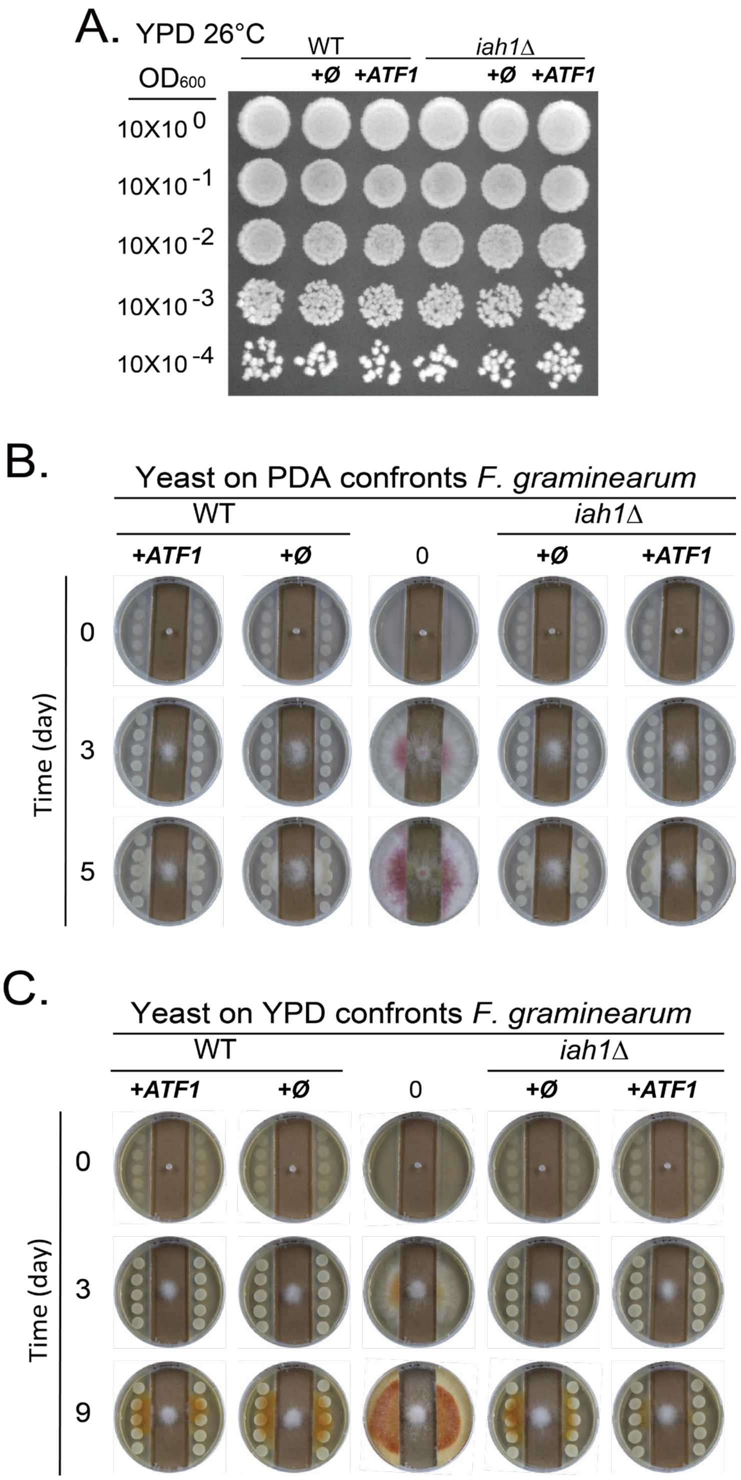

Figure S6. Detection of inhibition on *F. graminearum* by yeast VOCs from WT and *iah1*Δ strain with overexpression of *ATF1*. *ATF1* was overexpressed in WT and *iah1*Δ strain with a pUC19-*PGK1pro-ATF1-PGK1t-HIS3* plasmid. (**A**) There was no growth defect for overexpression of *ATF1* in either WT and *iah1*Δ strains on YPD medium at 26°C. Yeast cells at approximately 10 OD at the first row were serially diluted with a 10 × dilution. (**B**) Inhibitory effect on growth and differentiation of *F. graminearum* was weakly enhanced with overexpression of *ATF1* in WT and *iah1*Δ strain on PDA medium. Experiments were set up as in Figure 3A. (**C**) Inhibitory effect on growth and differentiation of *F. graminearum* was weakly enhanced with overexpression of *ATF1* in WT and *iah1*Δ strains on YPD medium. Experiments were set up as in Figure S2A. Results shown represent three independent experiments with three plates for each yeast initial amount in each experiment.
